# Case Report: Prominent Brainstem Involvement in Two Patients With Anti-CASPR2 Antibody-Associated Autoimmune Encephalitis

**DOI:** 10.3389/fimmu.2021.772763

**Published:** 2021-11-11

**Authors:** Pei Liu, Miao Bai, Chao Ma, Yaping Yan, Gejuan Zhang, Songdi Wu, Zunbo Li, Daidi Zhao, Kaixi Ren, Hongzeng Li, Jun Guo

**Affiliations:** ^1^ Department of Neurology, Tangdu Hospital, Air Force Medical University, Xi’an, China; ^2^ Department of Neurology, The First Hospital of Xi’an, Xi’an, China; ^3^ Department of Cardiology, Tangdu Hospital, Air Force Medical University, Xi’an, China; ^4^ College of Life Sciences, Shaanxi Normal University, Xi’an, China; ^5^ Department of Neurology, Xi’an No.3 Hospital, Xi’an, China; ^6^ Department of Neurology, Xi’an Gaoxin Hospital, Xi’an Medical College, Xi’an, China

**Keywords:** autoimmune, contactin-associated protein-like 2, encephalitis, brainstem, immunoglobulin G1

## Abstract

Anti-contactin-associated protein-like 2 (CASPR2) antibody-associated autoimmune encephalitis is commonly characterized by limbic encephalitis with clinical symptoms of mental and behavior disorders, cognitive impairment, deterioration of memory, and epilepsy. The classical lesions reported are located at the medial temporal lobe or hippocampus, whereas prominent brainstem lesions have not been addressed to date. Herein, we reported two patients mimicking progressive brainstem infarction with severe neurological manifestations. On brain magnetic resonance imaging (MRI), prominent brainstem lesions were noted, although multifocal lesions were also shown in the juxtacortical and subcortical white matters, basal ganglia, hippocampus, and cerebellar hemisphere. Unexpectedly and interestingly, both cases had detectable CASPR2 antibodies in sera, and an exclusive IgG1 subclass was documented in the further analysis. They were treated effectively with aggressive immunosuppressive therapies including corticosteroids, intravenous immunoglobulin G, and rituximab, with the first case achieving a rapid remission and the other undergoing a slow but gradual improvement. To the best of our knowledge, this is the first report on prominent brainstem involvement with definite MRI lesions in anti-CASPR2 antibody-associated autoimmune encephalitis, which helps to expand the clinical spectrum of this rare autoimmune disease and update the lesion patterns in the CNS.

## Introduction

Contactin-associated protein-like 2 (CASPR2) belongs to a distinct subgroup of the neurexin superfamily expressed at the distal part of the axon initial segment and in the juxtaparanodal region of nodes of Ranvier on myelinated axons of the peripheral nervous system (PNS) and the central nervous system (CNS) (1). It is demonstrated to form a cell adhesion complex with contactin-2 and cluster voltage-gated potassium channels (VGKC), and the complex is essential for the repolarization of the neuronal membrane following an action potential and mediating the balance between excitatory and inhibitory circuits (2). Anti-CASPR2 antibody, as part of the antibodies targeting VGKC, has been reported with various clinical manifestations based on the wide expression of CASPR2 in both CNS and PNS (3). As previously reported, the most common clinical syndrome associated with anti-CASPR2 antibody is autoimmune encephalitis, primarily limbic encephalitis with the presenting symptoms including mental and behavior disorders, cognitive impairment, deterioration of memory, and epilepsy. Besides, peripheral nerve hyperexcitability and Morvan’s syndrome are also reported with clinical manifestations of neuropathic pain, neuromyotonia, or autonomic dysfunction (3, 4). In recent years, the clinical spectrum of anti-CASPR2 antibody-associated disorders has been extensively expanded with the discovery of novel manifestations including parkinsonism (5), non-paraneoplastic cerebellar ataxia (4, 6, 7), Guillain–Barré syndrome (8), chorea (9), neuropathic pain (10), Creutzfeldt–Jakob disease (11), amyotrophic lateral sclerosis with frontotemporal dementia syndrome (12), orthostatic myoclonus (13), and eyelid tremor (14). In this study, we report for the first time two cases of anti-CASPR2 antibody-associated autoimmune encephalitis with prominent brainstem involvement, which may further expand the clinical profile of this rare autoimmune disease.

## Case Presentations

### Patient 1

In early February of 2021, a previously healthy 57-year-old man was admitted to the local hospital with complaints of progressive dizziness, binocular diplopia, and unsteady gait for 9 days after neck chiropractic. He also stated mild hemiplegia of the left leg and hemidysesthesia of the left hand. The patient reported a history of cigarette smoking for more than 30 years and denied the history of oral and genital ulceration, uveitis, skin lesions, and arthritis. The score of neurological function assessed by the National Institutes of Health Stroke Scale (NIHSS) was 3, including ataxia in the left upper and lower extremities and mild sensory loss in the left hand. Meanwhile, a modified Rankin scale (mRS) score of 2.0 was obtained based on the disability of daily living due to an unsteady gait. The brain magnetic resonance imaging (MRI) performed 9 days after onset showed multifocal lesions scattered in bilateral basal ganglia and midbrain, and some of them showed hyperintensities on diffusion-weighted imaging (DWI). The magnetic resonance angiography (MRA) of the brain was normal, and the carotid artery ultrasound was unremarkable. The patient was initially diagnosed as multiple cerebral infarctions and aspirin, clopidogrel, and statins were prescribed, but his clinical status continued to worsen. Then the patient was referred to our department from a local hospital for a further comprehensive assessment of his condition in early March.

On admission, the patient was well nourished with a body mass index (BMI) of 31.12 kg/m^2^. His neurologic examination showed the cognitive function was intact. The patient had fluent speech and was oriented. The visual acuity and visual field were intact, and pupil size was normal and well reactive to light. Moderate limitation of adduction in both eyes was observed, and there was no spontaneous or gaze-evoked nystagmus. The numbness of distal fingers in the left hand and a mild decrease of muscle strength in the left lower limb (BMRC grade 4) were noted. Deep tendon reflexes were exaggerated in the left limbs, and bilateral Babinski’s sign and the left Chaddock’s sign were positive. Notable dysdiadochokinesia was seen in the left limbs, together with left dysmetria in the finger–nose test and left instability in the heel–knee–tibia test. His gait was unsteady and Romberg’s test was positive. The aforementioned findings contributed to an increase of the mRS score to 3.

**Figure 1:**
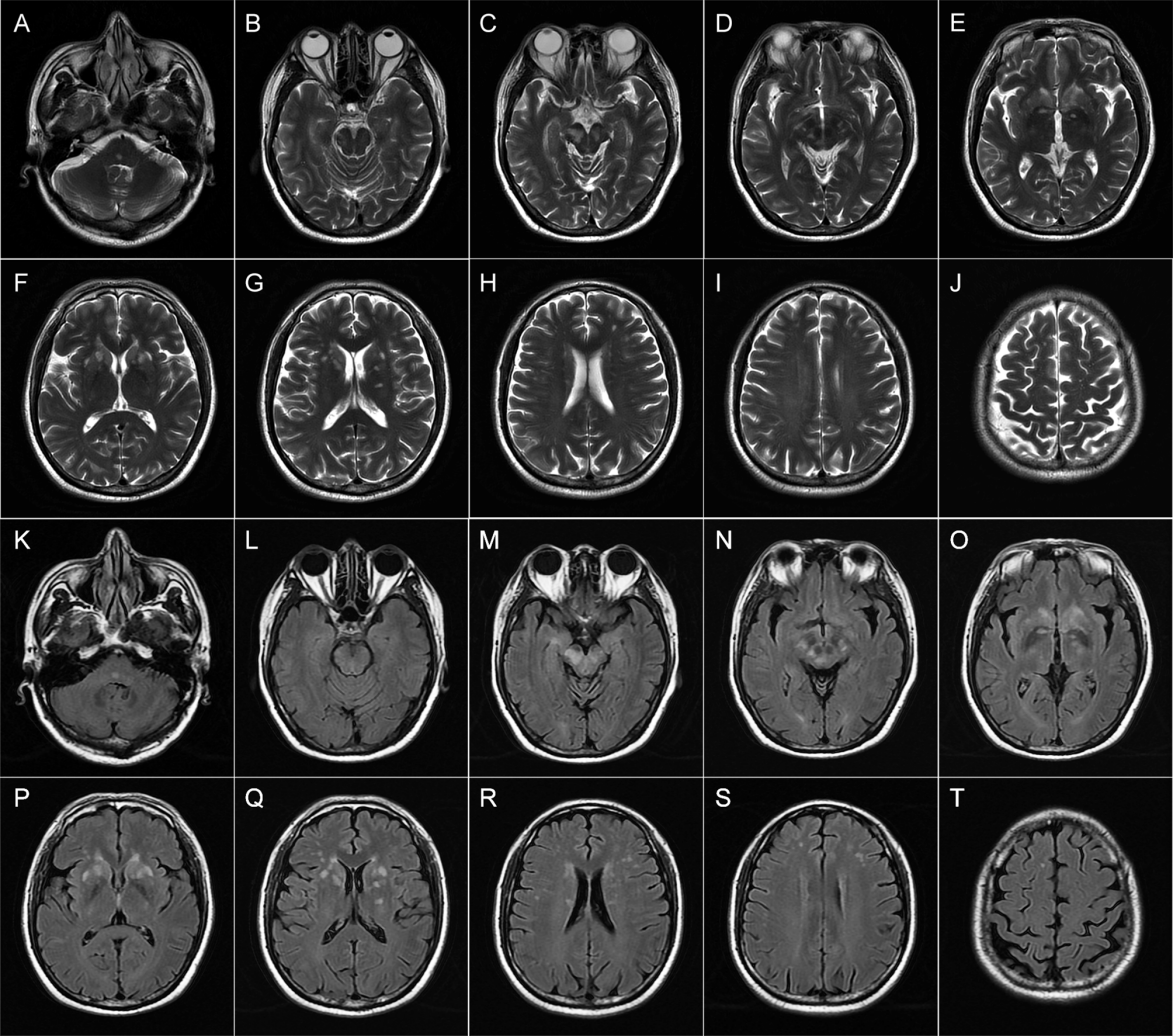
Brain magnetic resonance imaging (MRI) of patient 1 performed during acute attack. Axial T2-weighted **(A–J)** and FLAIR **(K–T)** images show multiple patchy hyperintense lesions in the tegmentum of the pons **(A, K)**, bilateral midbrain **(B–D, L–N)**, and right hippocampus **(C, M)**; ovoid lesions with well-defined borders in the bilateral head of the caudate nucleus and putamen **(E–G, O–Q)**; and spotty lesions involving bilateral paraventricular white matters **(H, R)** and subcortical white matters of the frontal lobes **(H–J, R–T)**.

Repeat brain MRI scan revealed multifocal T1 hypointensities and T2 and fluid-attenuated inversion recovery (FLAIR) hyperintensities scattered in the brainstem (midbrain and the tegmentum of pons), basal ganglia (head of caudate nucleus and putamen), right hippocampus, and paraventricular and subcortical white matters of bilateral frontal lobes (**Figure 1**). Some of these lesions showed hyperintensities on both DWI and apparent diffusion coefficient (ADC) maps and heterogeneous patchy or ring-like enhancement on gadolinium (Gd)-enhanced MRI (**Supplemental Figure 1**). Computerized tomography (CT) of the chest–abdomen–pelvis with and without contrast eliminated the presence of malignancies. The results of laboratory tests including complete blood count, urine and stool analyses, liver and kidney function, lipid profile, and blood glucose concentration were unremarkable. Serological tests for infections and autoimmune parameters were all normal. Cerebrospinal fluid (CSF) analysis revealed normal white blood cell count (0 × 10^6^/L), protein level (285.0 mg/L; normal range: 80–430 mg/L), and immunoglobulin G level (28.9 mg/L; normal range: 0–34 mg/L). No malignant lymphocytes were found in CSF. Metagenomic next-generation sequencing (mNGS) did not detect any pathogenic microorganisms in CSF. Three oligoclonal IgG bands (OCBs) were detected exclusively in CSF. Anti-CASPR2 antibodies with a titer of 1:10 were detected by cell-based assays (CBA) using HEK293 cells transfected with CASPR2 isoform 1 and confirmed by tissue-based assay (TBA) using rat brain tissue sections exclusively in serum but not in CSF. Further analysis *via* immunofluorescence with secondary antibodies against specific for IgG subclasses documented a subclass of IgG1 but not IgG2, IgG3, or IgG4. Other antibodies against NMDAR, LGI1, AMPAR1, AMPAR2, GABAAR-α1, GABAAR-β3, GABABR, Kelch-like protein 11, ganglionic AChR, mGluR1, mGluR5, D2R, Neurexin-3α, DPPX, IgLON5, GlyR-α1, AQP4, MOG, GFAP, Hu, Yo, Ri, CV2, Ma1, Ma2, SOX1, Zic4, GAD65, Tr/DNER, Titin, PKC-γ, Recoverin, and Amphiphysin were detected negative in both CSF and serum by CBA at the reference center (MYBiotech Co., Ltd., Xi’an, China). Based on the presence of specific anti-CASPR2 antibodies, a diagnosis of anti-CASPR2 antibody-associated autoimmune encephalitis was eventually established.

**Figure 2:**
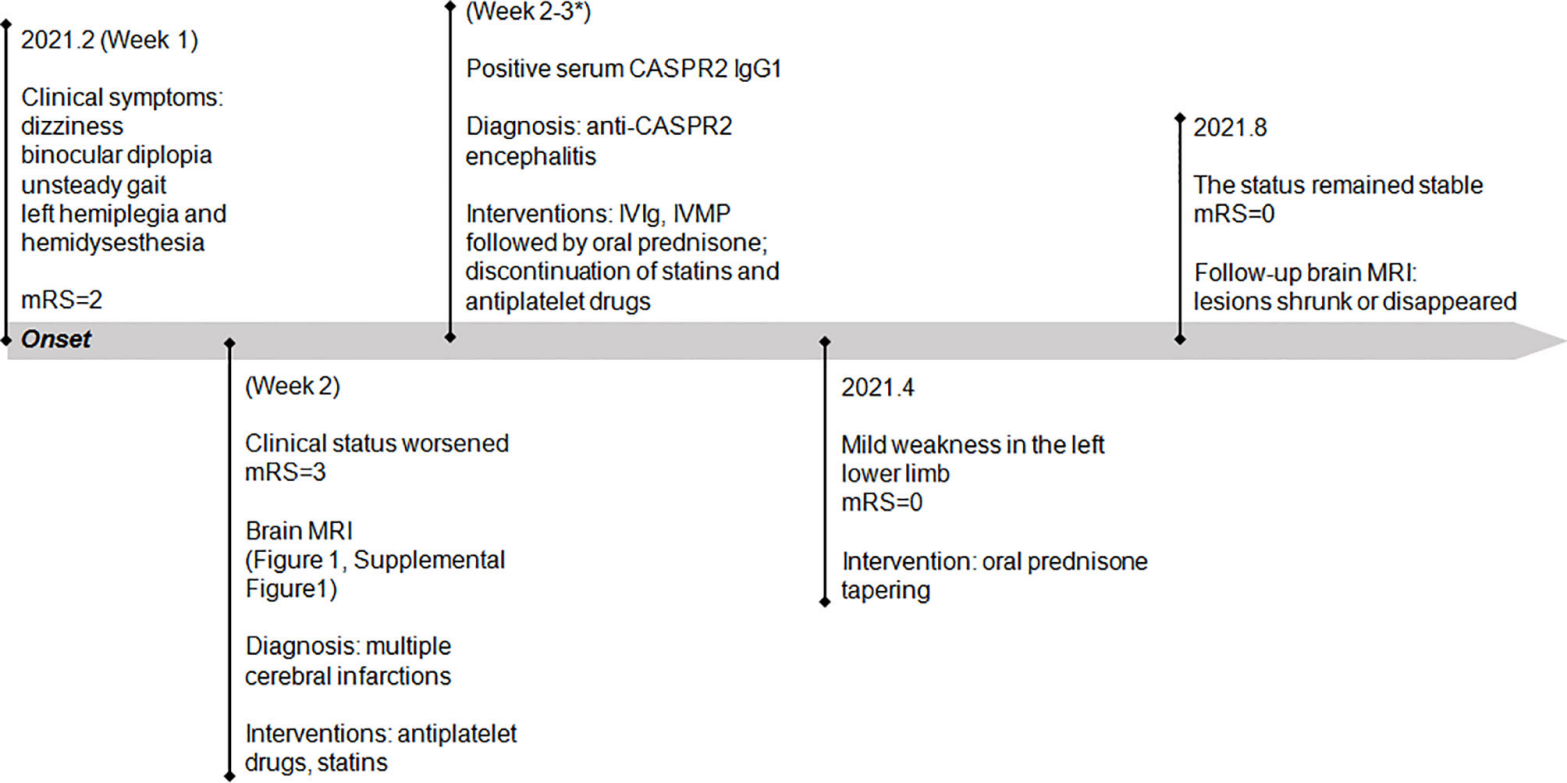
Timeline of patient 1 with relevant data of the episodes and interventions. *This admission. mRS, modified Rankin scale; IVIg, intravenous immunoglobulin; IVMP, intravenous methylprednisolone.

Statins and antiplatelet drugs were discontinued immediately, and the patient was then treated with intravenous immunoglobulin therapy (IVIg; 0.4 g/kg body weight for 5 consecutive days) plus high-dose intravenous methylprednisolone pulse therapy (1,000 mg/day for 3 days, 500 mg/day for 2 days) followed by oral prednisone at an initial dose of 40 mg daily with a slow tapering schedule of 5 mg every month. There was a significant improvement in diplopia and ataxia obtained 1 week after the initiation of immunotherapy. Then he continued to adhere to the intervention and was well tolerated. At 2-month follow-up visit after discharge, the patient reported that he had achieved complete remission of diplopia, ataxia, and numbness in the left hand, and only mild weakness in his left lower limb was left. Follow-up brain MRI revealed that the lesions had shrunk or disappeared. At the last follow-up in August, the status of the patient remained stable with the mRS score of 0. He reported that no adverse and unanticipated events occurred and was satisfied with the treatment that he received and the prognosis. The timeline of patient 1 with relevant data of the episodes and interventions is presented in **Figure 2**.

### Patient 2

In late May of 2021, a 55-year-old woman with a 6-month history of hypertension was admitted to the emergency department of a local hospital because of transient loss of consciousness followed by slurred speech, dysphagia, right hemianesthesia, and hemiparalysis (BMRC grade 4). No fever, headache, or abnormal mental behaviors were reported. There was no history of oral and genital ulceration and uveitis. Two months earlier, the patient had suffered from shingles with a rash on the right side of her face which had faded away before this admission. No fever, leukocytosis, and cutaneous edematous erythematous plaques were reported. After the possibility of intracranial hemorrhage was excluded by an urgent brain CT scan, acute cerebral infarction was suspected and the patient was treated with intravenous recombinant tissue plasminogen activator (rt-PA; 0.9 mg/kg body weight) followed by the administration of antiplatelet drugs and statins. However, the patient underwent rapid exacerbation of neurological impairments. The subsequent whole-brain digital subtraction angiography (DSA) revealed no arterial stenosis or aneurysm. To ascertain the underlying etiology, the patient was transferred to our department on day 4 after disease onset.

**Figure 3:**
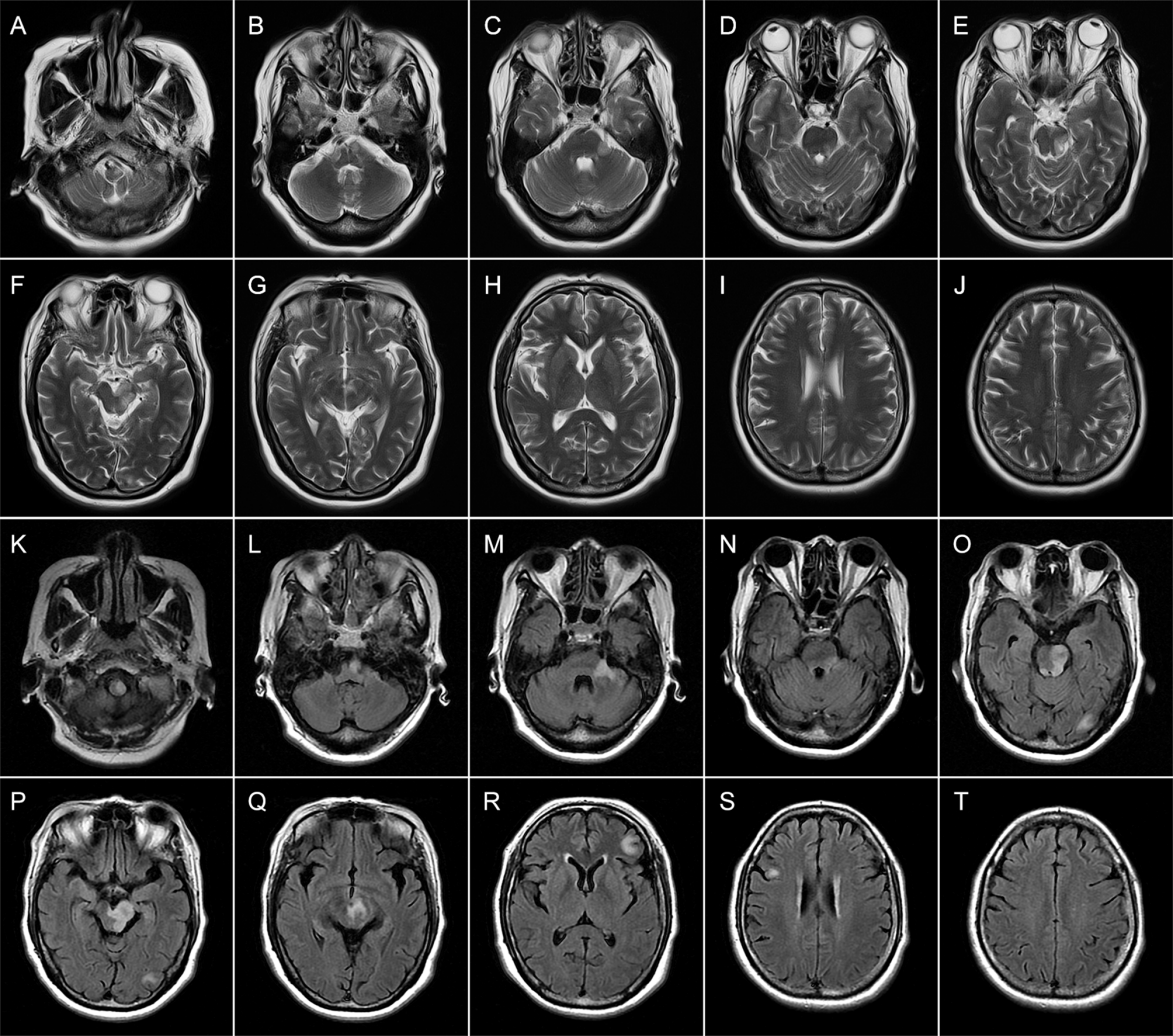
Brain magnetic resonance imaging (MRI) of patient 2 performed during acute attack. Axial T2-weighted **(A–J)** images obtained 4 days after onset show multiple patchy hyperintense lesions in the left brainstem (medulla, brachium pontis, and midbrain) **(A–G)** and juxtacortical white matters of the right frontal lobe **(I)**. Axial FLAIR **(K–T)** images obtained 15 days after onset reveal expanding lesions in the brainstem **(K–Q)**. Meanwhile, new patchy or spotty lesions are confirmed in the left occipital lobe **(O, P)** as well as juxtacortical and subcortical white matters of the left frontal lobes **(R, T)**.

**Figure 4:**
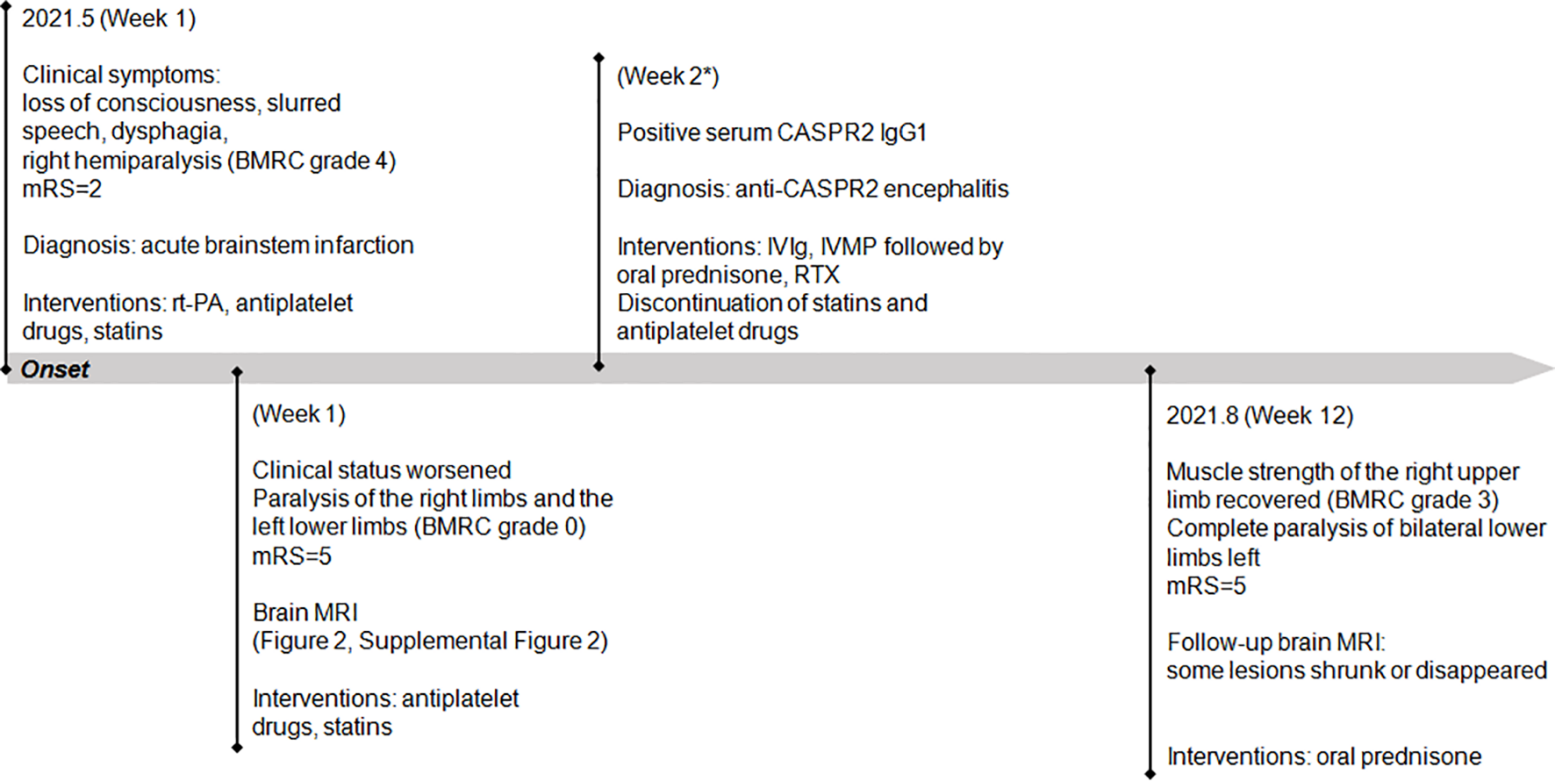
Timeline of patient 2 with relevant data of the past episodes and interventions. *This admission. mRS, modified Rankin scale; IVIg, intravenous immunoglobulin; IVMP, intravenous methylprednisolone; RTX, rituximab.

On admission, the vital signs of the patient were stable but with an increased blood pressure of 170/98 mmHg. On neurologic examination, she was in a lethargic state and unable to fully follow the orders of the clinician. Signs of left oculomotor nerve palsy, right central facial palsy, and bilateral true bulbar paralysis were noted. Meanwhile, there was complete paralysis of the right limbs and the left lower limbs, and muscle hypotonia was present in the corresponding limbs. Deep tendon reflexes were markedly diminished in all four limbs, and bilateral Babinski’s sign and Chaddock’s sign were positive. All the findings contributed to the mRS score of 5. Brain MRI showed multifocal T1 hypointensities and T2 and FLAIR hyperintensities in the left brainstem (medulla, brachium pontis, and midbrain) and juxtacortical and subcortical white matters of bilateral frontal lobes and the left occipital lobe (**Figure 3**). The majority of these lesions revealed hyperintensities on DWI and isointensities relative to normal white matter on ADC map (**Supplemental Figure 2**). All lesions did not show any enhancement on Gd-enhanced MRI. Consistent with the results obtained from prior whole-brain DSA, brain MRA did not find any cerebrovascular abnormalities. Chest–abdominal–pelvic CT excluded the possibility of malignancies. The results of routine laboratory tests including blood cell assay, erythrocyte sedimentation rate, and hypersensitive C-reactive protein were unremarkable. Serological tests for infections, malignancies, and autoimmune parameters were normal except for the presence of serum antinuclear antibody (ANA; titer 1:320). CSF analysis revealed mild leukocytosis (12 × 10^6^/L) and slightly elevated protein level (442.0 mg/L; normal range: 80–430 mg/L) as well as markedly elevated immunoglobulin G (69.4 mg/L; normal range: 0–34 mg/L) and interleukin-6 level (160.5 pg/ml; reference range: <7 pg/ml). Herpes simplex virus, Epstein–Barr virus, and human cytomegalovirus DNAs were undetectable, and no malignant lymphocytes were found in CSF. CBA with CASPR2 isoform 1-transfected HEK293 cells together with TBA using rat brain tissue sections confirmed the existence of anti-CASPR2 antibodies with a titer of 1:10 in serum but not in CSF, and further analysis *via* immunofluorescence with secondary antibodies against specific for IgG subclasses determined an exclusive IgG1 subclass. Other antibodies against NMDAR, AMPAR1, AMPAR2, LGI1, GABABR, DPPX, IgLON5, GlyR-α1, GABAAR-α1, GABAAR-β3, mGluR5, D2R, Neurexin-3α, GAD65, AQP4, MOG, and GFAP were detected negative in both serum and CSF by CBA at the reference center (MYBiotech Co., Ltd., Xi’an, China). The patient was diagnosed as anti-CASPR2 antibody-associated autoimmune encephalitis and treated with intravenous immunoglobulin therapy (IVIg; 0.4 g/kg body weight for 5 consecutive days) combined with intravenous methylprednisolone pulse therapy (1,000 mg/day for 3 days, 500 mg/day for 3 days, 240 mg/day for 3 days, 120 mg/day for 3 days) followed by oral prednisone at an initial dose of 50 mg daily with a slow tapering schedule of 5 mg every 3 weeks. Initial intravenous immunotherapy controlled the progression of this disease, but no obvious improvement was achieved. Thereafter, induction therapy with rituximab was given at 100 mg per week for 3 consecutive weeks, and then the patient underwent a slow but gradual improvement of clinical symptoms. Repeat brain MRI was performed at 1 month after discharge and some lesions had shrunk or disappeared. She was well tolerated and stated that she would continue to adhere to the intervention. The maintenance rituximab at 100 mg would be infused every 6 months as planned, and follow-up is still ongoing. The timeline of patient 2 with relevant data of the episodes and interventions is presented in **Figure 4**.

## Discussion

As is well known, the detection of anti-CASPR2 antibodies in serum or CSF is crucial for the definite diagnosis of anti-CASPR2 antibody-associated disorders (3), whereas in recent years, the extensive expansion of clinical spectrum has made it more difficult to differentiate this disease from other mimics, in particular when anti-CASPR2 antibodies cannot be tested or the results have not been obtained (4, 15). Similarly, both cases in this study mimicked progressive brainstem infarction with severe neurological manifestations, rather than presented with characteristic limbic encephalitis or refractory seizures. Acute-onset stroke-like presentation and unilaterally dominant brainstem lesions might be the main causes of the initial misdiagnosis made by the local clinicians. However, no imaging manifestations suggestive of cytotoxic edema based on hyperintensities on DWI but without restricted diffusion on the ADC map, no evidence of intracranial vascular stenosis, and more notably, no response to the treatment of cerebral infarction and even gradual exacerbation of clinical symptoms led us to explore the possibility of alternative diagnoses. We excluded major inflammatory and immune-mediated disorders prone to brainstem lesions such as multiple sclerosis, neuro-Behçet disease, neuro-Sweet syndrome, neurosarcoidosis, chronic lymphocytic inflammation with pontine perivascular enhancement responsive to steroids (CLIPPERS), Susac syndrome, and malignant lymphoma, mainly based on previous history, clinical features, and MRI clues of the brainstem lesions (16). Then a thorough screening of antibodies targeting neuronal and glial antigens was carried out. Unexpectedly and interestingly, anti-CASPR2 antibodies were detected in the sera of both cases, which contributed to the determination of the final diagnosis. To the best of our knowledge, this is the first report that showed prominent brainstem involvement with definite MRI lesions in anti-CASPR2 antibody-associated autoimmune encephalitis, which emphasized the necessity of thorough investigations including autoimmune parameters in patients with progressive brainstem involvement that cannot be explained by ischemic infarction.

Robust imaging evidence reveals that the medial temporal lobe is the most vulnerable CNS region associated with anti-CASPR2 antibodies (4, 15, 17, 18). In a review enrolling 667 patients with anti-CASPR2 antibody-associated disorders, 299 received MRI scans and abnormal CNS lesions were reported in 159 cases. Of these, 67 (42.1%) presented with encephalitis or T2 hyperintensities in the medial temporal lobes, occupying the first place of the affected CNS regions in anti-CASPR2 antibody-associated autoimmune encephalitis (4). Similar imaging findings have been demonstrated in a recent multicenter cohort study conducted in a Chinese population, in which the medial temporal lobe or hippocampus can be affected unilaterally or bilaterally (18). In addition, regional hypometabolism or hypermetabolism in the medial temporal lobes or basal ganglia was revealed by fluorodeoxyglucose positron emission tomography (FDG-PET), also indicating the involvement of these regions (4, 18). Inconsistent with previous studies, brain MRI did not find remarkable lesions in the medial temporal lobes of the present two patients; instead, multifocal lesions involving white and gray matters were noted in other regions, particularly in the brainstem. Of note, brainstem lesions associated with anti-CASPR2 antibodies have not been reported to date. In a prior study, bilateral brainstem abnormalities were proven by brainstem acoustic evoked potentials (BAEPs) and were well responsible for new-onset treatment-refractory respiratory failure in a 68-year-old male with anti-CASPR2 antibody-associated autoimmune encephalitis; however, no remarkable lesions were revealed by brain MRI scan and Gd-enhanced sequences (19). Meanwhile, we retrospectively reviewed the brain MRI results of other 14 patients with anti-CASPR2 antibody-associated autoimmune encephalitis from three centers in Northwest China between June 2018 and June 2021, and no definite brainstem lesions were found in all cases (data not shown). Collectively, the brainstem is a rare but not negligible vulnerable region in anti-CASPR2 antibody-associated autoimmune encephalitis, and in the future, the incidence of brainstem lesions needs to be estimated in a large patient population.

It is well demonstrated that anti-CASPR2 antibodies may result in CNS involvement, PNS involvement, or both. However, notably distinct patterns of antibody distribution have been reported in patients with different clinical phenotypes. When PNS was involved solely or together with CNS, anti-CASPR2 antibodies could be detected exclusively in the sera of all patients but not in CSF (2, 20). In contrast, these antibodies were detectable in both sera and CSF of the patients with pure CNS involvement (2, 4, 20), with the former exhibiting much higher antibody titers and detectable rate than the latter (2, 17, 20). In this study, both patients had positive anti-CASPR2 antibodies in serum but not in CSF, which is consistent with previous case reports (21–23). A similar pattern of antibody distribution was observed in other 11 patients with anti-CASPR2 antibody-associated autoimmune encephalitis from three centers from Northwest China, where all cases had positive serum antibodies but only five cases had positive CSF results (data not shown). This is possibly due to the predominant initial activation of the peripheral immune system without intrathecal autoantibody synthesis (10), and CSF might also have detectable anti-CASPR2 antibodies during the subsequent longitudinal antibody monitoring.

To date, all four IgG subclasses, i.e., IgG1–4, have been reported in anti-CASPR2 antibody-associated disorders (17), but central and peripheral clinical phenotypes appear to have a different predominance of antibody subclasses especially for IgG1 and IgG4, even though controversial results were obtained (2, 10, 20, 24). In a retrospective study involving 22 patients with a high likelihood of anti-CASPR2 encephalitis, 18 had positive serum IgG1 subclass with the highest prevalence. Of these, 13 also had IgG2, 2 IgG3, and 16 IgG4, and CSF subclasses were subset of those in serum (17). In contrast, another retrospective cohort study enrolling 17 patients with anti-CASPR2 autoimmune encephalitis found that CSF IgG4 subclass was much more common than IgG1 subclass with the proportion of 100% and 58.8%, respectively, and the two subclasses have similar proportions in serum (91.7% vs. 100%) (20). Till now, the reported pathophysiology of anti-CASPR2 antibody-associated encephalitis seems to be inconsistent, and it is speculated that it depends to some extent on the subclasses of predominant antibodies. Anti-CASPR2 IgG1 antibodies have been demonstrated to initiate a destructive, complement-mediated process leading to irreversible neuronal loss and subsequent brain tissue atrophy especially in the hippocampus (10), whereas non-complement-activating IgG4 antibodies showing low affinities for Fc gamma receptors inhibit the interaction of CASPR2 with contactin-2 but do not cause internalization of CASPR2 (24). In this study, we analyzed serum anti-CASPR2 antibody subclasses as well and found the exclusive IgG1 in both cases, which suggests that complement-activating IgG1 subclass might dominate in contributing to CNS lesions. Thus far, the mechanisms responsible for the propensity of brainstem involvement in our cases remain unclear. It is possible that the predominant exposure of antigenic epitopes in this specific region facilitates the preferential binding of anti-CASPR2 IgG1 to the target self-antigens and triggers subsequent pathogenic cascades. This hypothesis needs to be verified in future studies, for instance, those with animal models passively induced by anti-CASPR2 antibodies from serum or CSF samples of patients with prominent brainstem involvement and pathological studies in this unique patient subgroup. Moreover, long-term follow-up is warranted to determine whether brainstem atrophy would occur later.

At present, there are no standard recommendations or consensus-based practice guidelines for the treatment of anti-CASPR2 antibody-associated autoimmune encephalitis, and available data from large retrospective and observational studies are sparse. However, given the autoimmune participation in this disease entity, early and aggressive therapies focusing on different segments of immune responses have been applied in several studies and good/full or partial responses can be achieved in the majority of patients (10, 15). The first-line immunotherapies consist of corticosteroids, IVIg, plasma exchange (PE), or combined treatment. Other second-line immunosuppressive agents such as azathioprine, mycophenolate, rituximab, and cyclophosphamide could be considered when no favorable responses to sufficient first-line therapies are obtained (3, 15). In this study, one case had full neurologic recovery after corticosteroids (intravenous and oral) plus IVIg were given, whereas the other did not have a favorable response to the first-line therapies including corticosteroids and IVIg. Then a low-dose rituximab therapeutic strategy was administered because of good efficacy, safety, and tolerance of this regimen in treating neuromyelitis optica, myasthenia gravis, and NMDAR encephalitis in our previous clinical practice. Encouragingly, the case achieved a slow but gradual neurologic improvement, and long-term follow-ups are still on schedule for the evaluation of her clinical status. Meanwhile, dynamic monitoring and screening of neuronal and glial antibodies are required to explore the possibility of the coexistence of anti-CASPR2 antibodies and merging novel antibodies such as Kelch-like protein 11.

## Conclusions

Anti-CASPR2 antibody-associated autoimmune encephalitis is a rare disease entity with variable imaging abnormalities on brain MRI. Brainstem involvement with definite MRI lesions may serve as the initial presentation and the most prominent characteristic during disease, which expands the clinical spectrum of this disorder. Autoantibody detection is crucial for definite diagnosis and differentiation from other mimics. Analysis of IgG subclasses would help give insights into the potential pathogeneses and provide valuable suggestions for treatment strategy options.

## Data Availability Statement

The raw data supporting the conclusions of this article will be made available by the authors, without undue reservation.

## Ethics Statement

The studies involving human participants were reviewed and approved by the Ethics Committees of Tangdu Hospital, Air Force Medical University (approval number: TDLL-KY-202108-03). The patients/participants provided their written informed consent to participate in this study.

## Author Contributions

PL drafted the manuscript. PL, MB, and CM collected and interpreted the data. YY performed the antibody detection experiments. GZ, SW, and ZL provided multicenter data. DZ and KR prepared the figures. HL and JG conceived and designed this study. JG critically revised the final manuscript. All authors contributed to the article and approved the submitted version.

## Funding

This study was supported by the Science and Technology Innovation and Development Foundation of Tangdu Hospital (grant number 2019LCYJ010) and the Excellent Personnel Foundation of Tangdu Hospital in 2021. The funders had no role in the study design, data collection and interpretation, or the decision to submit the work for publication.

## Conflict of Interest

The authors declare that the research was conducted in the absence of any commercial or financial relationships that could be construed as a potential conflict of interest.

## Publisher’s Note

All claims expressed in this article are solely those of the authors and do not necessarily represent those of their affiliated organizations, or those of the publisher, the editors and the reviewers. Any product that may be evaluated in this article, or claim that may be made by its manufacturer, is not guaranteed or endorsed by the publisher.
