# Supplementary material for: Case Report: Prominent Brainstem Involvement in Two Patients With Anti-CASPR2 Antibody-Associated Autoimmune Encephalitis
Source: Front Immunol. 2021 Nov 11;12:772763. doi: 10.3389/fimmu.2021.772763 (PMC8631873; doi:10.3389/fimmu.2021.772763)
Supplement: Supplementary file 1 [file DataSheet_1.pdf]

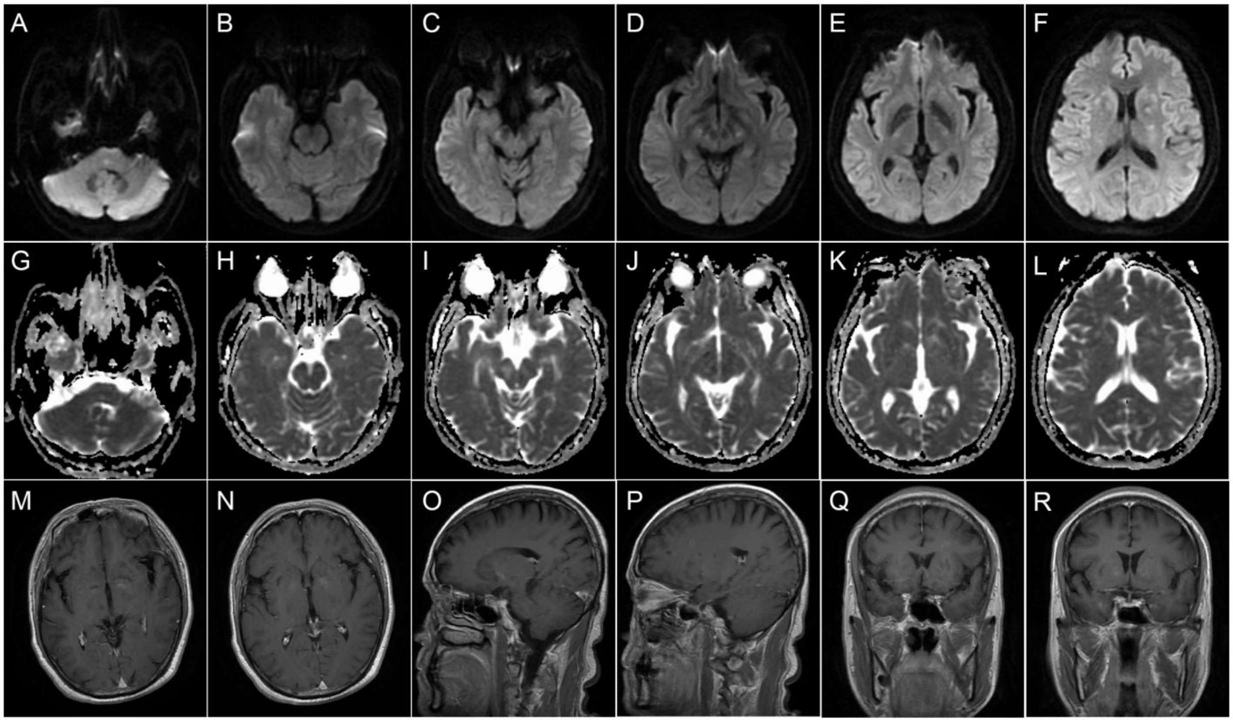

SUPPLEMENTAL FIGURE 1 Brain diffusion-weighted imaging (DWI), apparent diffusion coefficient (ADC) map and Gd-enhanced magnetic resonance imaging (MRI) of patient 1 performed during acute attack. Axial images (A–L) show multiple hyperintensities on both DWI (A–F) and ADC (G–L) maps located in the pons (A, B, G, H), bilateral midbrain (C, D, I, J) and the basal ganglia (E, F, K, L). Axial (M, N), sagittal (O, P) and coronal (Q, R) Gd-enhanced images show heterogeneous patchy or ring-like enhancement in the lesions scattered in bilateral basal ganglia (M–R) and deep white matter of the left frontal lobe (Q).

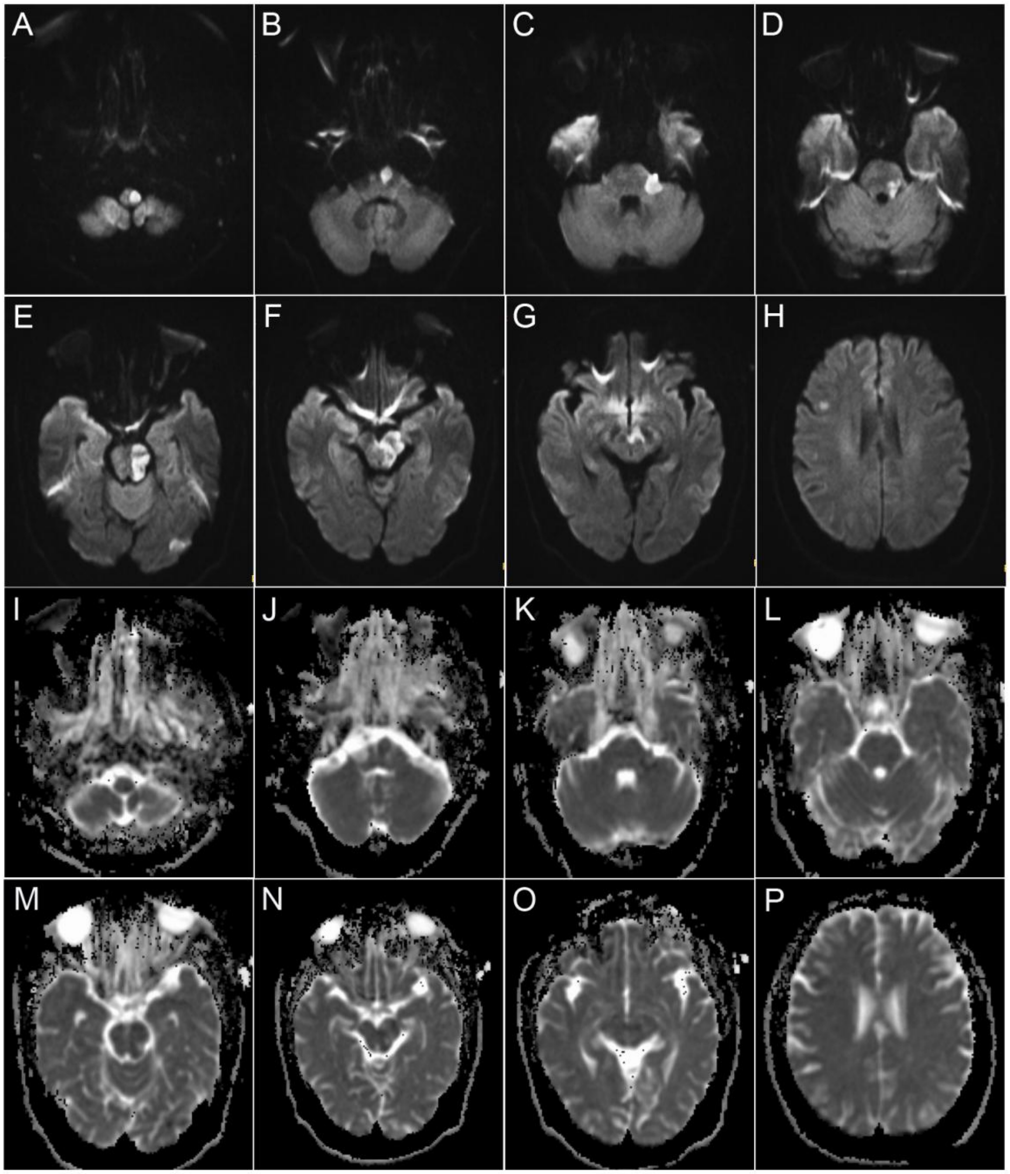

SUPPLEMENTAL FIGURE 2. Brain diffusion-weighted imaging (DWI) and apparent diffusion coefficient (ADC) maps of patient 2 performed during acute attack (15 days after onset). Axial (A–P) images show multiple hyperintensities on DWI (A–H) and isointensities relative to normal white matter on ADC (I–P) maps located in the left medulla (A, B, I, J), brachium pontis (C, D, K, L), the left pontine (E, M) and midbrain (F, G, N, O) as well as juxtacortical white matters of the right frontal lobe (H, P).
